# The TGF-β/Smad Repressor TG-Interacting Factor 1 (TGIF1) Plays a Role in Radiation-Induced Intestinal Injury Independently of a Smad Signaling Pathway

**DOI:** 10.1371/journal.pone.0035672

**Published:** 2012-05-02

**Authors:** Mohammad Hneino, Agnes François, Valerie Buard, Georges Tarlet, Rym Abderrahmani, Karl Blirando, Pamela A. Hoodless, Marc Benderitter, Fabien Milliat

**Affiliations:** 1 Laboratory of Radiopathology and Experimental Therapies, Institute for Radiological Protection and Nuclear Safety (IRSN), Fontenay-aux-Roses, France; 2 Terry Fox Laboratory, British Columbia Cancer Agency and the University of British Columbia, Vancouver, British Columbia, Canada; National Cancer Institute, United States of America

## Abstract

Despite advances in radiation delivery protocols, exposure of normal tissues during the course of radiation therapy remains a limiting factor of cancer treatment. If the canonical TGF-β/Smad pathway has been extensively studied and implicated in the development of radiation damage in various organs, the precise modalities of its activation following radiation exposure remain elusive. In the present study, we hypothesized that TGF-β1 signaling and target genes expression may depend on radiation-induced modifications in Smad transcriptional co-repressors/inhibitors expressions (TGIF1, SnoN, Ski and Smad7). In endothelial cells (HUVECs) and in a model of experimental radiation enteropathy in mice, radiation exposure increases expression of TGF-β/Smad pathway and of its target gene PAI-1, together with the overexpression of Smad co-repressor TGIF1. In mice, TGIF1 deficiency is not associated with changes in the expression of radiation-induced TGF-β pathway-related transcripts following localized small intestinal irradiation. In HUVECs, TGIF1 overexpression or silencing has no influence either on the radiation-induced Smad activation or the Smad3-dependent PAI-1 overexpression. However, TGIF1 genetic deficiency sensitizes mice to radiation-induced intestinal damage after total body or localized small intestinal radiation exposure, demonstrating that TGIF1 plays a role in radiation-induced intestinal injury. In conclusion, the TGF-β/Smad co-repressor TGIF1 plays a role in radiation-induced normal tissue damage by a Smad-independent mechanism.

## Introduction

Radiation therapy is one of the most important methods of curing cancer and more than half of cancer patients will receive radiotherapy at some stage during the course of their treatment. New radiotherapy techniques aim to improve the radiation dose gradient to the target tissues and to reduce the dose to the surrounding normal tissues at risk. However, radiation-induced normal tissue toxicity remains a major contributor to complication after cancer therapy [1]. Radiation injury is a complex process that occurs in organized tissues orchestrated by mutually dependent cellular lineages as well as a multitude of biologically active molecules such as growth factors, cytokines and chemokines [2], [3].

The Transforming Growth Factor-β1 (TGF-β1) is a highly important growth factor among the panel of molecules expressed in tissues after radiation exposure and TGF-β1 contribution in the initiation and progression of radiation-induced normal tissue damage is well documented [4], [5]. Several studies have demonstrated a positive correlation between the severity of radiation injury and TGF-β1 signal activation. Radiation-induced up-regulation of TGF-β1 expression levels has been shown in lung [6], kidney [7], intestine [8], skin [9], liver [10], bladder [11] and rectum [12]. In addition to TGF-β1 itself, several studies have shown up-regulation of intracellular effectors of TGF-β1 signaling and, in particular, the canonical Smad-dependent pathway [12]–[14]. TGF-β1 transduces signals by binding to complexes of type I (ALK1 and ALK5) and type II serine/threonine kinase receptors. Ligand binding induces phosphorylation of the receptors, which then activate intracellular signaling via phosphorylation of receptor R-Smads (Smad2 and Smad3). R-Smad then complex with Smad4, triggering nuclear translocation and subsequent regulation of expression of specific target genes [15]. Increased expression of the TGF-β1 receptors, ALK5 and endoglin [13], [16], Smad mediators, Smad2 and Smad3 [14], [17], and increased expression of TGF-β/Smad target genes such as Plasminogen Activator Inhibitor type 1 (PAI-1) and Connective Tissue Growth Factor (CTGF) [13], [14], [18] were reported after irradiation *in vivo* and *in vitro*. Moreover, skin damage is reduced in Smad3^−/−^ mice following ionizing radiation exposure, suggesting that Smad signalling contributes to radiation-induced injury [19], [20].

If TGF-β1 has been demonstrated to be a key mediator involved in radiation-induced normal tissue damage, the mechanism by which the TGF-β/Smad signal is activated is not fully understood. Ubiquitous expression of TGF-β1 imposes a fine tuning of signal transduction. Smad-mediated signals induced by TGF-β1 are tightly regulated by negative-feedback mechanisms via inhibitory Smads (I-Smads) [21] and Smad co-repressors such as c-Ski, SnoN and TG-Interacting Factor 1 (TGIF1) [22], [23]. I-Smads (i.e. Smad6 and Smad7) can inhibit the activation of R-Smads by competing for type I receptor interaction, and by recruiting specific ubiquitin ligases, known as Smad ubiquitination regulatory factor (Smurfs), or phosphatases to the activated receptor complex, thereby targeting it for proteosomal degradation or dephosphorylation [24]. Recentlty, particular attention was paid for the smad co-repressor TGIF1 for its expanding biological roles in development [25], [26], acute myeloid leukemia [27], and hematopoietic cell proliferation and differentiation [28]. TGIF1 is a member of the three aminoacid loop extension (TALE) class of homeodomain proteins that functions both as a co-repressor of the TGF-β1 pathway and as a competitor of the retinoic acid pathway [29]. TGF-β1-dependent transcriptional repression by TGIF1 is mediated by direct competition with the coactivator p300/CBP for Smad2 interaction, thereby repressing TGF-β1 activated genes. In addition and independently of TGF-β1 signaling, TGIF1 exerts intrinsic transcriptional repression by direct DNA binding through the recruitment of the histone deacetylase complex, the carboxyl-terminus binding protein complex, and the Sin3 complex, all of which modify chromatin configuration and limit the transcription of several target genes [30].

Thus, several recent studies in mouse models of renal fibrosis strongly suggest that down-regulation of Smad7 or Smad co-repressors Ski and SnoN expressions leads to an amplification of TGF-β signaling, which contributes to the progression of inflammation and fibrosis [31]–[34]. In the present study, we hypothesized that TGF-β1 signaling and downstream target genes could be influenced by modifications of I-Smad and/or Smad co-repressors expression in response to irradiation. We demonstrate *in vivo* in a model of radiation-induced small intestinal damage in mice and *in vitro* in a model of endothelial cells that radiation-induced sustained activation of TGF-β/Smad pathway is not related to the down regulation of its negative feedback mechanisms. Moreover, we show that TGIF1 plays a role in radiation-induced small intestinal damage independently of the Smad pathway.

## Methods and Materials

### Animals and Irradiation Procedure

C57BL/6 mice (named wild type) were purchased from Charles River Laboratories, TGIF1 knockout mice were maintained on a C57BL/6J background as previously described thus TGIF1^+/+^, TGIF1^+/−^ and TGIF1^−/−^ were from the same colony [35]. Genotype was determined by PCR screening (Figure S1). For total body irradiation (TBI), TGIF1^+/+^, TGIF1^+/−^ and TGIF1^−/−^ mice (n = 10 to 12 mice per group) were anesthetized with i.p. injection of a ketamine/xylazine solution and were exposed to 13 Gy, a radiation dose known to induce gastrointestinal syndrome, using a ^60^Cobalt source (dose rate 1.4 Gy.min^−1^). Localized intestinal radiation injury was performed by exposure of an intestinal segment to 19 Gy of radiation. Briefly, TGIF1^+/+^, TGIF1^+/−^ and TGIF1^−/−^ mice were anesthetized with isoflurane and a 3-cm-long intestinal segment (10 cm from the ileocecal valve) was exteriorized and exposed to a single dose of 19 Gy (^60^Co source dose rate 1.2 Gy/minute). Sham irradiation was performed by maintaining the intestinal segment exteriorized without radiation exposure. After radiation exposure or sham irradiation, the exposed segment was returned to the abdominal cavity and peritoneum/abdominal muscles and skin were separately closed with interrupted sutures and mice were euthanized at different times after irradiation. For survival curves, mice were followed until a clinical “limit point” in accordance with ethic guidelines, i.e. humanely euthanized when presenting clinical signs of distress such as prostration, severe weight loss and dyspnea. For TBI survival curves, mice were monitored twice a day, included week-ends.

Following localized small intestinal irradiation, intestinal segments from sham and irradiated mice were harvested and snap-frozen in liquid nitrogen and stored at −80°C for RNA isolation. All experiments were conducted in compliance with legal regulations in France for animal experimentation, and protocols were approved by the ethics committee for animal experimentation of the Institute for Radiological Protection and Nuclear Safety (number P05-09).

### Histology and Immunohistochemistry

Five-micrometer-thick sections of mouse small intestinal tissue were stained with hematoxylin-eosin-saffron for routine histology. Morphometric analyses were performed using the Visiol@b™ 2000 image analysis software (Biocom SA, Les Ulis, France). For each animal, villus height value is the mean of 20 villi measured. The intraindividual variation was about 10%. Data are presented as the mean values derived from between 3 and 6 tissue sections from different mice per group ± standard error of the mean. Crypt number was assessed along 1000 µm of longitudinal section. Data are presented as mean values derived from between 3 and 6 tissue sections from different mice per group ± standard error of the mean.

Specimens of normal tissue from patients treated for rectal adenocarcinoma with preoperative radiotherapy (45 Gy; 2 or 1.8 Gy by fraction) were taken from the irradiated field adjacent to the tumor and from microscopically normal mucosa distant from the tumor (about 6 weeks after the last fraction). Formalin-fixed, paraffin-embedded tissue samples were obtained following institutional ethics (Gustave Roussy Institute) and French Medical Research Council guidelines. No consent was required and sections from tissues patients were obtained retrospectively several years after surgery and data were analyzed anonymously. Briefly 5-µm sections were incubated with anti-TGIF1 (sc-17800, Santa Cruz Biotechnology, Heidelberg, Germany). Biotinylated rabbit anti-mouse IgG and streptavidin/biotinylated-peroxidase kit (DAKO) were used before revelation by DAB substrate kit (Dako, Trappes, France) and counterstained with hematoxylin.

### Endothelial Cell Culture and Irradiation

Human umbilical vascular endothelial cells (HUVECs) were purchased from Lonza (Verviers, Belgium) and cultured in EGM-2-MV medium at 37°C with 5% CO2. Cells were used between passages 4 and 5 and were irradiated at 2, 10 and 20 Gy with a ^137^Cesium source.

### RNA Isolation, Reverse Transcription and Real-time PCR

Total RNA was prepared with the total RNA isolation kit (Rneasy Mini Kit, Qiagen, Valencia, CA). RNA quantification and integrity was analysed using Agilent 2100 bioanalyzer. RT was performed with 1 µg RNA using a reverse transcription kit from Applied Biosystems (Courtaboeuf, France). Pre-developed TaqMan gene expression assays (Applied Biosystems) were used to quantify transcript levels of all studied genes (see Table S1). qPCR was carried out using the ABI PRISM 7900 Sequence detection system, and the results were normalized to the housekeeping genes GAPDH or 18S for respectively *in vitro* or *in vivo* experiments. Relative mRNA was quantified using the ΔΔC_T_ method.

### Transient Transfection and Reporter Gene Assay

HUVECs were transiently co-transfected with (CAGA)9-Lux reporter or PAI- Luc plasmids together with myc-Smad3 or myc-TGIF using FuGENE HD (Roche Diagnostics, Meylan, France). In all experiments, co-transfection with renilla luciferase pRLTK vector was used for normalization. 24 hours after transfection, cells were serum depleted for 18 hours and were irradiated and/or treated with 10 ng/mL of TGF-β1. The cells were lysed 24 hours later and relative luciferase activity was measured using a Mithras luminometer (Berthold Technologies, Bad Wildbad, Germany) by the Dual-Luciferase reporter assay according the manufacturer’s instructions (Promega, Charbonnières, France).

### Western Blot Analysis

Proteins lysed in RIPA buffer supplemented with proteases and phosphatases inhibitors were separated by SDS-polyacrylamide gel electrophoresis before transfer onto nitrocellulose membranes. The following protein-specific primary antibodies were used: anti-TGIF1 (sc9825), anti-Ski (sc-33693), anti SnoN (sc-9141), anti-Myc (9E10) (Santa-Cruz Biotechnology), anti-Smad7 (MAB2029, R&D Systems Europe, Lille, France), anti-PAI-1 (Novocastra Laboratories Ltd., Newcastle, UK), anti-Smad3 (Zymed), anti-phospho-Smad3 (C25A9, Cell signaling technology, Danvers, MA). Membranes were incubated with HRP-conjugated secondary antibody (Amersham, Orsay, France) and were developed using enhanced chemiluminescence (Amersham). Membranes were stripped and reprobed with anti-glyceraldehyde-3-phosphate dehydrogenase (GAPDH) antibody (Biodesign, Saco, ME) to detect GAPDH expression as a loading control.

### TGIF Gene Silencing using siRNA

Human TGIF1 siRNA and non-targeting negative control siRNA (ON-TARGETplus SMARTpool TGIF1 L-011404 and ON-TARGETplus Non-targeting Pool) were purchased from Dharmacon (Chicago, IL). HUVECs were seeded into 6 well plates for 24 h to reach 50–70% confluence and were transfected with siRNA at a final concentration of 100 nM using Dharmafect according to the manufacturer's protocols. The medium was replaced 24 h after transfection and cells were irradiated and processed for further analysis. The silencing efficiency of siRNAs was confirmed by real time PCR and western blot analyses.

### Statistical Analyses

Data are given as means ± SEM. Statistical analyses were performed by ANOVA or Student’s t test with a level of significance of P<0.05. Mouse survival curves were calculated by the Kaplan-Meier method and compared using the log rank test.

## Results

### Overexpression of Molecular Actors of TGF-β/Smad Signaling in Mouse Radiation Enteropathy is Associated with Increased Expression of Smad co-repressors TGIF1 and SnoN

**Figure 1:**
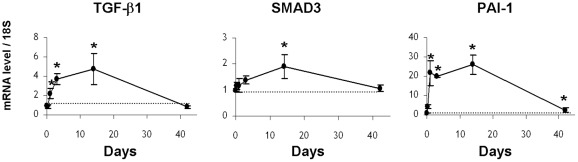
Radiation induces activation of the TGF-β pathway in a mouse model of 19 Gy-localized small intestinal irradiation: mRNA levels measured by real time PCR of TGF-β1, SMAD3 and PAI-1 in total intestinal tissues, 5 hours, 1 day, 3 days, 14 days and 42 days after irradiation. mRNA level of 18S was used as housekeeping gene and dot line indicates value 1 assigned for control sham-operated animals at each time. Results are +/− SEM (n = 6 to 8 mice/group). *p<0.05 versus sham mice.

**Figure 2:**
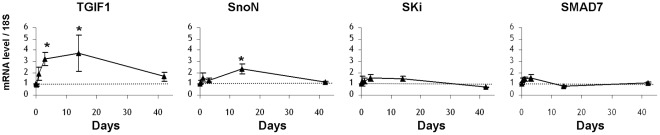
Kinetic analyses of mRNA levels of SMAD co-repressors and inhibitors in a mouse model of 19 Gy-localized small intestinal irradiation: mRNA levels of TGIF1, SnoN, Ski and SMAD7 were measured by real time PCR in total intestinal tissues, 5 hours, 1 day, 3 days, 14 days and 42 days after irradiation. mRNA level of 18S was used as housekeeping gene and dot line indicates value 1 assigned for control sham-operated animals at each time. Results are +/- SEM (n = 6 to 8 mice/group). *p<0.05 versus sham mice.

We used an *in vivo* model of radiation enteropathy to investigate the kinetics of mRNA levels of TGF-β1 itself, an effector of Smad signaling, Smad3, and a TGF-β1/Smad-target gene, PAI-1. During the observation period (days 1, 3, 14 and 42 after irradiation), mRNA levels of TGF-β1, Smad3 and PAI-1 increased progressively to reach a peak at 14 days, compared to control levels in sham-operated mice for each time point (Figure 1). Expression of TGF-β1 and Smad3 decreased to control levels by 42 days, whereas PAI-1 expression was still 2.5 times greater compared to the control level (Figure 1). These observations provide strong evidence that our model is appropriate to study a putative implication of Smad co-repressors and inhibitors in the radiation-induced TGF-β/Smad activation pathway. To investigate whether TGF-β-signaling pathway activation in mouse irradiated intestine could be related to aberrant regulation of Smad transcriptional co-repressors/inhibitors, (ie TGIF1, SnoN, Ski and Smad7), kinetic analysis of their transcript levels in irradiated intestinal tissues was performed. Figure 2 shows that the kinetics of TGIF1 and SnoN gene expression follows the same pattern of variation as TGF-β1, Smad3 and PAI-1 mRNA levels. No variations were observed for Ski and Smad7. In our model of radiation enteropathy, up-regulation of TGF-β signaling transcripts (TGF-β1 and Smad3) and the TGF-β1 downstream target gene PAI-1 is associated with an increased expression of the Smad co-repressors TGIF1 and SnoN.

### Expression of TGF-β/Smad Regulatory Elements and PAI-1 in a Radiation Dose - and Time-dependent Manner in Endothelial Cells

**Figure 3:**
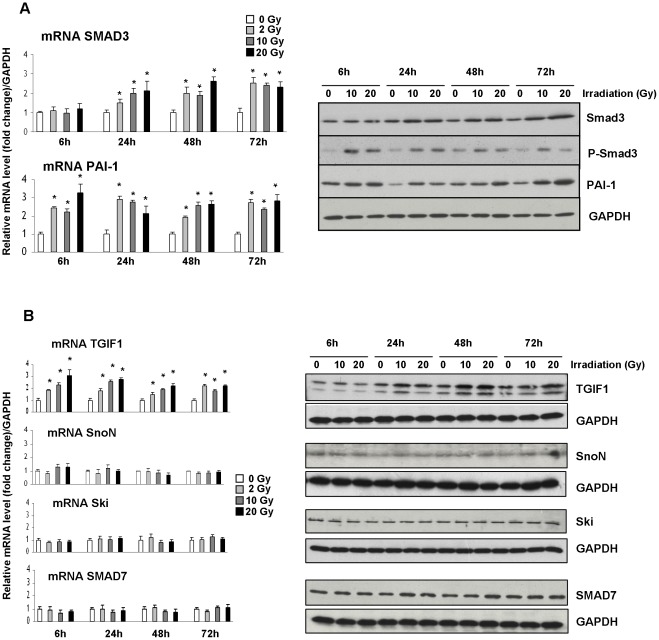
Effect of irradiation on mRNA and protein expression of SMAD3, PAI-1 and SMAD co-repressors and inhibitors in HUVEC. (A) Irradiation induces SMAD pathway and PAI-1 in HUVEC cells. mRNA levels of SMAD3 and PAI-1 in HUVEC 6, 24, 48 and 72 hours after irradiation were measured by real time PCR. mRNA level of GAPDH was used as housekeeping gene and value 1 was assigned to unirradiated cells for each time. Protein levels of Smad3, phospho-Smad3 and PAI-1 were followed by western blot. (B) Radiation dose- and time-dependent mRNA level and protein abundance of TGIF1, SnoN, Ski and Smad7 were measured by real time PCR and western blot. Results are the mean ± SEM of 3 independent experiments realized in triplicates. *P<0.05 vs unirradiated cells.

Previous studies from our group showed that radiation-induced rectal damage in humans is associated with overexpression of PAI-1 and phospho–Smad2/3 in the endothelium [14]. TGIF1 immunohistochemical staining performed on human rectum sections revealed a positive immunoreactivity in the smooth muscle layers and endothelium of submucosal vessels (Figure S2). Even if it was not possible to ascertain differences in TGIF1 staining intensity between unexposed and irradiated tissues in the endothelial compartment, this observation comforted us in the choice of human endothelial cells for *in vitro* studies. We then used Human Umbilical Endothelial Cells (HUVECs) to investigate whether the expression of TGF-β/Smad co-repressors and inhibitors is modulated in response to ionizing radiation. Time-course and dose-response experiments in HUVECs revealed that Smad3 and PAI-1 transcript levels, as well as their protein abundance, increased after irradiation (figure 3A). Moreover, the phosphorylated form of Smad3 increased rapidly after irradiation, demonstrating an activation of TGF-β/Smad pathway signaling in this *in vitro* model. Radiation exposure did not affect Ski, SnoN, and Smad7 mRNA and protein expression in either a dose- or time- dependent manner. Interestingly, and in accordance with *in vivo* results, after irradiation TGIF1 expression increased at both the mRNA and protein levels (figure 3B), suggesting that TGIF1 could affect the normal tissue response to ionizing radiation.

### TGIF1 Genetic Deficiency Sensitizes Mice to Radiation-induced Intestinal Damage

**Figure 4:**
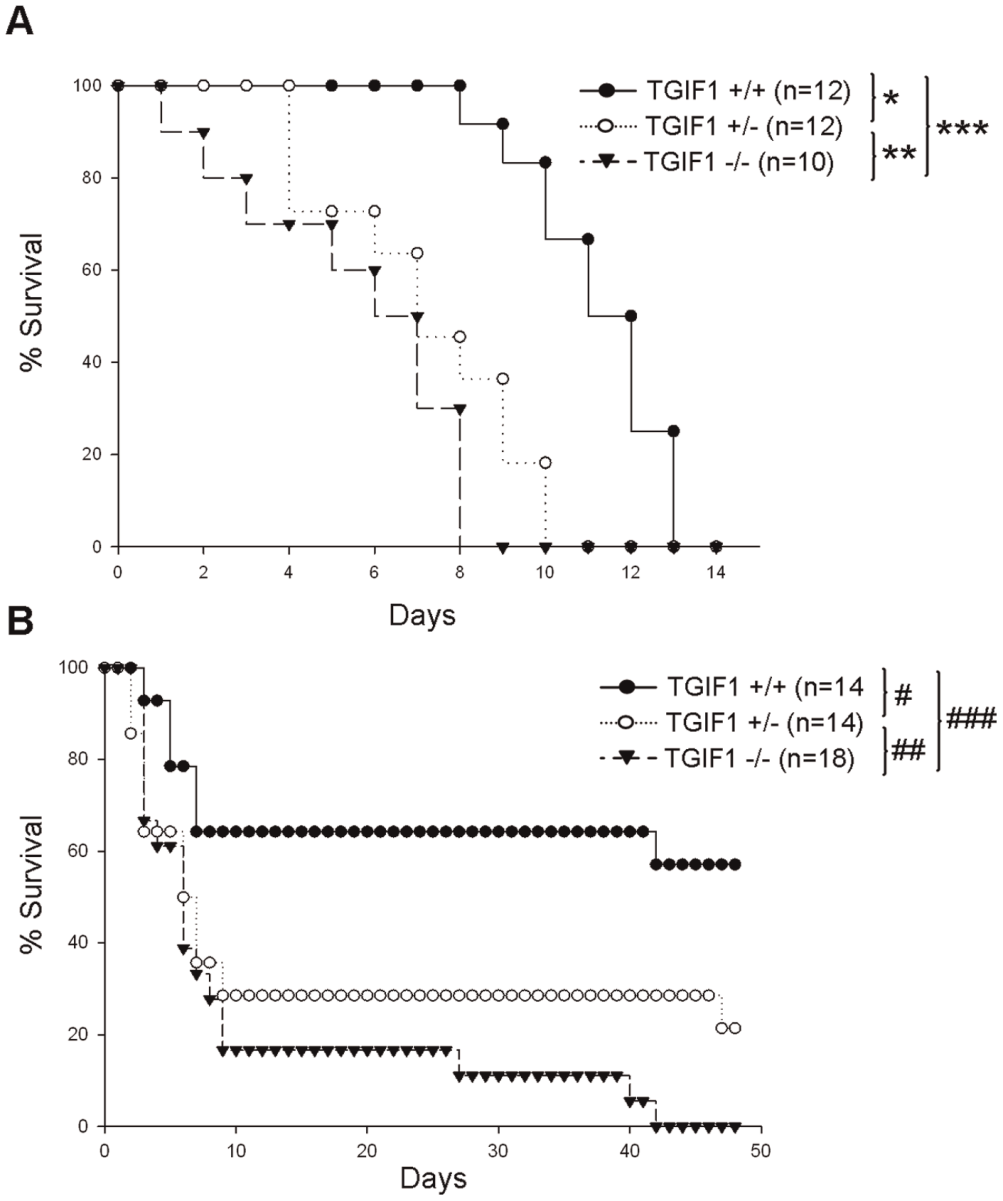
TGIF1 genetic deficiency sensitizes mice to gastrointestinal syndrome and radiation enteropathy. Kaplan-Meier analyses represent the percent survival of TGIF1^+/+^, TGIF1^+/−^ and TGIF1^−/−^ mice following (A) 13 Gy total body irradiation and (B) 19 Gy localized small intestinal irradiation. Statistical differences were determined by the log rank test, *p = 0.0033, **p = 0.19, ***p = 0.00065, ^#^p = 0.06, ^##^p = 0.11, ^###^p = 0.000014.

To prove the involvement of TGIF1 in radiation-induced intestinal damage, we followed the survival of TGIF1 deficient mice under two irradiation conditions: a radiation–induced lethal gastrointestinal syndrome after 13 Gy total body irradiation (TBI), and localized radiation-induced enteropathy after 19 Gy irradiation. After a lethal 13 Gy TBI, Kaplan-Meier curves show that homozygous and heterozygous TGIF1 deficient mice died more rapidly than TGIF1^+/+^ mice. Eight days after irradiation, no TGIF1^−/−^ mice survived whereas 100% lethality in TGIF1^+/−^ and TGIF1^+/+^ mice occurred at 10 and 13 days, respectively (figure 4A). After a 13 Gy TBI and without bone marrow transplantation, the cause of death is due to both bone marrow destruction and gastrointestinal damage. Survival curves obtained after TBI irradiation suggest that TGIF1 plays a role in radiation-induced gastrointestinal damage, but one may not exclude a role for TGIF1 *via* the bone marrow compartment in this model. In the model of localized radiation enteropathy, Kaplan-Meier analyses show that 40% of TGIF1^+/+^ died rapidly in the first 10 days (figure 4B). At day 10, lethality is more pronounced in TGIF1^+/−^ (70%) and TGIF1^–/–^ (84%) mice. Fifty days after irradiation, no TGIF1^−/−^ mice survived whereas 58% of TGIF1^+/+^ and 21% of TGIF1^+/−^ were still alive (figure 4B). Three days after 19 Gy localized small intestinal irradiation, histological examination (Figure S3) revealed mucosal inflammatory infiltrate (A) and significant crypt loss in all tissues (B). Crypt loss was significantly more severe in TGIF1^−/−^ mice than in other groups. No significant change in villus height was observed at this time point in any group (C).

### TGIF1 Deficiency is not Associated with Changes in Radiation-induced TGF-β Pathway-Related Transcripts in Mouse Radiation Enteropathy

**Figure 5:**
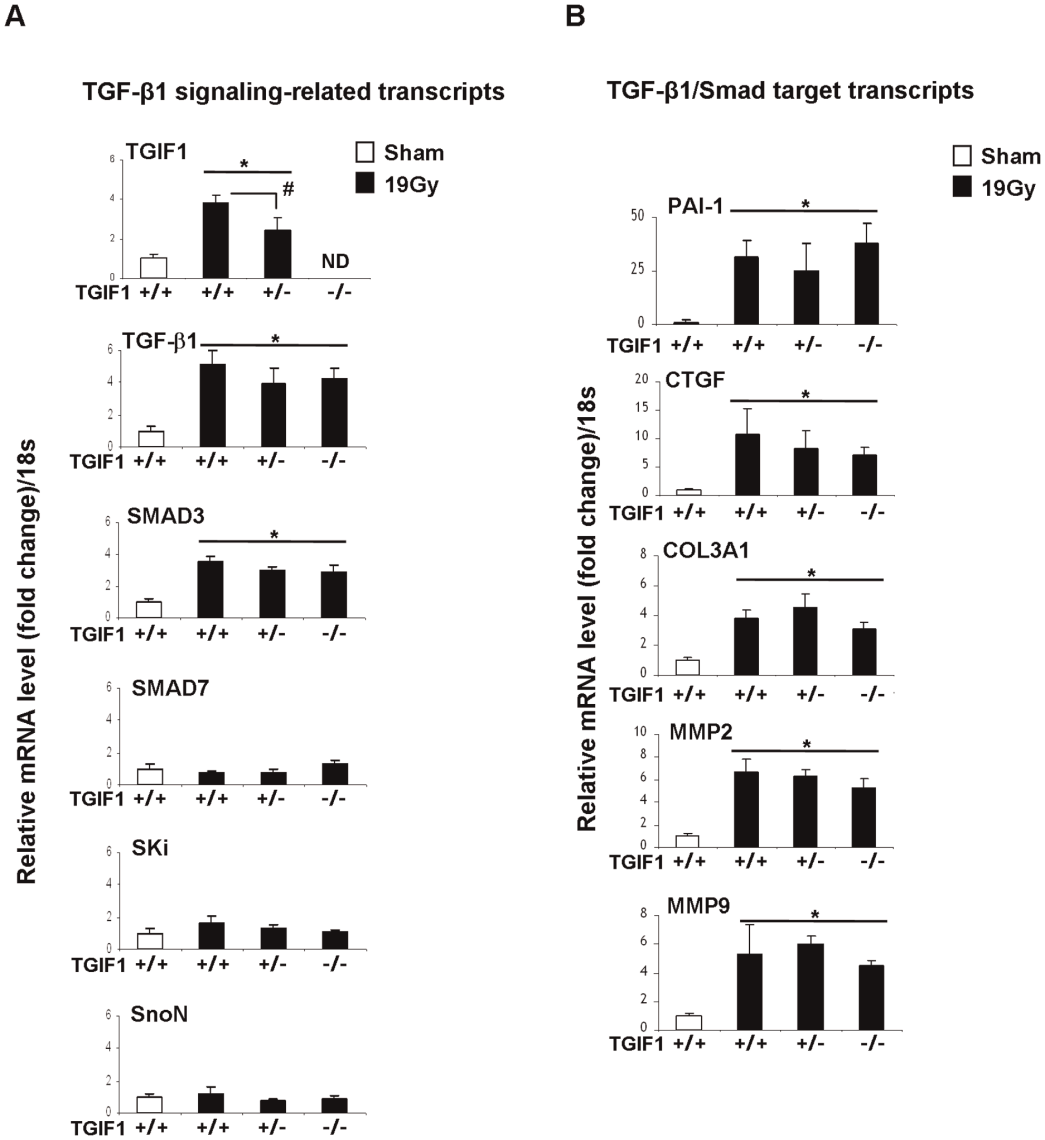
TGIF1 genetic deficiency is not associated with modifications of radiation-induced TGF-β pathway-related molecular profile *in vivo*. mRNA levels of (A) TGF-β signaling-related genes and (B) TGF-β/Smad target genes in TGIF1^+/+^, TGIF1^+/−^ and TGIF1^−/−^ mice 3 days after 19 Gy localized small intestinal irradiation were determined by real time PCR. mRNA level of 18S was used as housekeeping gene and value 1 was assigned to sham-operated TGIF1^+/+^ animals. Results are the mean ± SEM with 8 to 10 mice per group. *P<0.05 vs TGIF1^+/+^ sham mice. ^#^P<0.05 vs TGIF1^+/+^ irradiated mice. No significant difference was obtained between TGIF1^+/+^, TGIF1^+/−^ and TGIF1^−/−^ sham-irradiated animals.

TGIF1-deficient mice showed an enhanced sensitivity to TBI and localized intestinal radiation exposure, thus we examined whether the exacerbated radiosensitivity of TGIF1 deficient mice is associated with a deleterious role of TGF-β/Smad pathway signaling activation in radiation intestinal damage. We studied the acute effect of 19 Gy localized intestinal radiation on TGF-β/Smad signaling-related transcripts (TGIF1, TGF-β1, Smad3, Smad7, Ski and SnoN) and Smad-target gene-related transcripts (PAI-1, CTGF, COL3AI, MMP2 and MMP9) at day 3 in TGIF1^+/+^, TGIF1^+/−^ and TGIF1^−/−^ mice. Consistent with our previous data, irradiation increased expression of TGF-β1, Smad3 and TGIF1. No significant variation was observed for Smad7, ski and SnoN (figure 5A). In addition, comparisons of mRNA expression profiles of intestinal sections of irradiated TGIF1^−/−^ and TGIF1^+/−^ versus irradiated TGIF1^+/+^ mice showed that TGIF1 deficiency did not alter radiation-induced TGF-β1 and Smad3 expression. In a similar fashion, we showed that the lack of TGIF1 did not modify the radiation-induced up-regulation of TGF-β/Smad target genes such as PAI-1, CTGF, COL3AI, MMP2 and MMP9 (figure 5B). All together, these results suggest that the influence of TGIF1 on radiation-induced intestinal sensitivity *in vivo* is independent of the TGF-β/Smad canonical pathway.

### Radiation-induced TGF-β/Smad Pathway Activation in HUVECs is Independent of TGIF1

**Figure 6:**
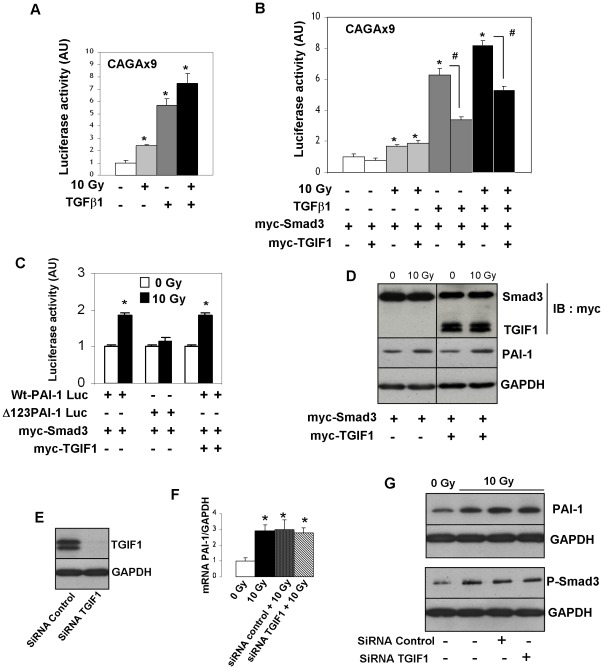
TGIF1 has no effect either on radiation-induced Smad pathway activation or on radiation-induced PAI-1 overexpression in endothelial cells. (A) HUVECs were transfected with (CAGA)9-Luc reporter plasmid for 24 h and were serum starved for 18 hours. Cells were irradiated at 10 Gy in presence or not of 10 ng/mL TGF-β1. Relative luciferase activity was measured 24 h after irradiation (B) HUVECs were co-transfected for 24 h with CAGA9-Lux and myc-Smad3 with or without myc-TGIF1. Relative luciferase activity was measured 24 h after irradiation and/or treatment with 10 ng/mL of TGF-β1. (C) HUVECs were transfected with wt-PAI-1 Luc reporter or CAGA box-mutated PAI-1 Luc reporter (Δ123-PAI-1 Luc) and myc-Smad3 with or without myc-TGIF1. Luciferase activity was assayed 24 hours after irradiation. Relative luciferase activity is the mean ± SEM of at least 3 independent experiments realized in triplicates (D) HUVECs were transfected for 24 h with myc-Smad3 with or without myc-TGIF1. Cells were irradiated at 10 Gy and PAI-1 expression was measured 24 h after irradiation. E-G) HUVECs were transfected with non–targeting siRNA (siRNA control) or siRNA TGIF1 for 24 h and irradiated at 10 Gy. Silencing efficiency was confirmed by western-blot (E). mRNA level (F) and protein levels of PAI-1 and Phospho-Smad3 were performed by western-blots (G). Results are the mean +/- SEM of two independent experiments realized in triplicates and representative blots from three independent experiments are shown. *p<0.05

By using the TGF-β/Smad responsive gene reporter vector (CAGA9-luc), radiation-induced Smad-dependent transcription was measured in HUVECs after 10 Gy exposure in the presence or absence TGF-β1. Results showed that both irradiation and TGF-β1 activate Smad-dependent transcription in HUVECs (figure 6A). Next, we investigated the impact of TGIF1 overexpression on TGF-β1 - and radiation-induced Smad transcription. Luciferase activity in HUVECs transfected with myc-Smad3, with or without myc-TGIF1, and with or without treatment with TGF-β1 and/or irradiation, showed that overexpression of TGIF1 represses the TGF-β-induced SMAD-dependent transcriptional activation but not the radiation-induced SMAD-dependent transcription (figure 6B). To confirm these results, we examined whether TGIF1 overexpression affects radiation-induced Smad-dependent transcription of PAI-1. A PAI-1 promoter-luciferase reporter assay was performed in HUVECs co-transfected with myc-Smad3 with or without myc-TGIF1, and with Wt-PAI-Luc gene reporter vector or CAGA box-mutated PAI-1 Luc gene reporter (Δb123-PAI-1 Luc). Radiation increased luciferase activity in HUVECs transfected with Wt-PAI-1-luc but not with Δb123-PAI-1 Luc. Radiation-induced, Smad-dependent PAI-1 transcription was not influenced by TGIF1 over-expression (Figure 6C). Western blot experiments performed on HUVECs cotransfected with myc-Smad3 with or without myc-TGIF1 confirmed that TGIF1 overexpression has no effect on radiation-induced PAI-1 protein expression (figure 6D). To complete these results, TGIF1 expression knock-down was performed using TGIF1 siRNA in HUVECs to explore the impact of TGIF1 silencing on radiation-induced Smad3 and PAI-1 expressions. Silencing efficiency (90%) was confirmed by real time PCR (data not shown) and western blot (figure 6E). TGIF1 knock down had no effect on radiation-induced mRNA levels of Smad3 and PAI-1, and had no effect on either PAI-1 protein level or the phosphorylated form of Smad3 (figure 6F and G).

## Discussion

Increased expression, secretion, and activation of TGF-β1 and Smad following radiation exposure [13], [14], [36]–[38] strongly suggests that TGF-β/Smad-dependent signaling is involved in the initiation and progression of radiation-induced normal tissue injury. In irradiated tissues, TGF-β1 cytokine production may result from transcriptional, translational and/or post translational mechanisms [37], [39], [40]. In addition to TGF-β1 itself, irradiation also induces the up-regulation of TGF-β receptors, activation of Smad2/3, and the subsequent increase in expression of Smad target genes, such as PAI-1 and CTGF, in different *in vivo* and *in vitro* models [13], [14], [17], [38]. Despite all of these extended studies of TGF-β signaling activation after irradiation, the role of Smad inhibitors and co-repressors has never been investigated in the context of radiation-induced normal tissue injury.

Here we show for the first time that the Smad co-repressor TGIF1 plays a role in radiation-induced intestinal damage and, unexpectedly, we demonstrate that radiation-induced Smad signaling in endothelial cells is independent of TGIF1.

On the basis of previous studies showing that downregulation of Smad inhibitors and Smad co-repressors amplifies TGF-β signaling activation and contributes to the progression of fibrosis [31], [32], we hypothesized that Smad co-repressors and I-Smad down-regulation could be responsible in part for radiation-induced activation of TGF-β signaling and downstream target genes expression. In our experimental model of mouse radiation enteropathy, activation of the pro-fibrosing TGF-β/Smad pathway and its target gene PAI-1 is associated with increased expressions of two Smad co-repressors, mainly TGIF1 and to a lesser extend SnoN.

To investigate the *in vitro* expression of TGF-β/Smad co-repressors and inhibitors in response to ionizing radiation, we chose endothelial cells for two reasons: 1) for the positive immunoreactivity of TGIF1 phospho-Smad2/3 and PAI-1 in the endothelium of submucosal vessels of human irradiated normal rectum and 2) for the activation of TGF-β signaling in response to irradiation previously shown by our group and others [14], [38]. Kinetic analysis of transcriptional levels of Smad co-repressors/inhibitors in HUVECs shows an increase in TGIF1 expression after irradiation. Our *in vivo* and *in vitro* results show that TGIF is a radiation target gene. We then showed that TGIF1 genetic deficiency in mice enhanced radiation sensitivity in cases of either TBI or localized high dose intestinal radiation exposure, demonstrating the principle that TGIF1 modulation affects the normal tissue response to ionizing radiation.

Proliferation and apoptosis are two crucial events in the intestinal tissue response to high dose radiation exposure. TGIF1 genetic deficiency is associated with the deregulation of fibroblast proliferation and progression through the G1 cell cycle phase [35], and down-regulation of TGIF1 sensitizes HepG2 cells to arsenic trioxide-induced apoptosis [41]. Changes in cell proliferation and/or apoptosis in radiosensitive compartments could explain the enhanced radiosensitivity of TGIF1 deficient mice. TGIF1 deficient irradiated intestine does not show any detectable variation in the expression of the principle G1 cell cycle regulatory genes (*i.e.* p21, cylin D, p27 and p15) (data not shown). However, crypt loss is more severe in TGIF1^−/−^ mice than in other groups and may reflect either increased radiation-induced cell loss and/or reduced regenerative cell proliferation. The measure of the apoptotic index of intestinal epithelial cells and microvascular endothelial cells will be a promising continuation of this work.

TGIF1 exerts several different modes of TGF-β/Smad dependent transcriptional repression. TGIF1 replaces the co-activators that bind to Smad2 and Smad3, and also recruits other general co-repressors, including histone deacetylases (HDACs) and Sin3, to the complex, thus repressing TGF-β/Smad target genes [30]. In addition, TGIF1 may also regulate ubiquitin-dependant degradation of Smad2 [42], and by recruiting carboxyl-terminus binding protein, can also repress TGF-β1 gene transcription via interactions with polycomb group proteins [43]. We examined whether the exacerbated radiosensitivity of TGIF1 deficient mice is associated with the deleterious role of TGF-β/Smad signaling activation reported in diverse irradiated tissues. Analyses after 19 Gy localized intestinal irradiation reveals that TGIF1 deficiency does not alter the expression of either radiation-induced TGF-β1 signaling gene expression (TGF-β1, Smad3, Smad7, Ski and SnoN) or radiation-induced TGFβ/Smad target gene (PAI-1, CTGF, COL3AI, MMP2 and MMP9). These *in vivo* results suggest that TGIF1 exerts a role in radiation-induced injury without altering Smad target gene transcription. *In vitro*, overexpression of TGIF1 in HUVECs represses TGF-β1-induced SMAD-dependent transcriptional activation, showing that TGIF1 is a key regulator of TGF-β1 signaling in endothelial cells. However, TGIF1 overexpression affects neither the radiation-induced Smad-dependent transcription, nor the radiation-induced Smad-dependent PAI-1 overexpression. In support of these results, TGIF1 silencing in HUVECs has no effect on radiation-induced PAI-1 expression. Altogether, our *in vivo* and *in vitro* results show that TGIF1 plays a role in radiation-induced injury independently of a Smad signaling pathway. Moreover, our experiments reveal differences between the TGF-β1 and the radiation-induced Smad-dependent transcription in endothelial cells, and molecular mechanisms involved in these differences will have to be elucidated.

Moreover, TGIF1 has been reported to interact with Ras/MAPKinase, ERK1/2 and NF-κb pathway and thereby contributes to inflammation, differentiation and apoptosis in response to a variety of growth factors (EGF and HGF) and cytokines (TNF-α) [44]–[46]. Irradiation activates MAPKinase and NF-κb pathways in different tissues and in endothelial cells [47], [48], and it would be interesting to explore whether TGIF1 genetic deficiency is associated with a deregulation in one or more of these pathways that could contribute to the sensitization of TGIF1 deficient mice to radiation damage.

In conclusion, this is the first report which shows that TGIF1 is increased in response to ionizing radiation, and that this protein plays a role in radiation injury. The molecular mechanism by which TGIF1 is involved in radiation-induced intestinal damage seems to be independent of TGF-β/Smad signaling and future investigations to understand the role of TGIF1 in radiation-induced injury need to be performed.

## Supporting Information

Figure S1 **Example of PCR screening for TGIF1 genotype.**
(TIF)

Figure S2 **Representative microscopic images from immuno-labelling of TGIF1 on human rectal tissue submucosal vessels.** (A) unirradiated tissue; (B) dystrophic vessel in the irradiated area from the same patient. Magnification x 200.
(TIF)

Figure S3 **Radiation-induced small intestinal damage.** (A) Images of small intestinal tissues obtained 3 days after sham or 19 Gy localized small intestinal irradiation in wild type, TGIF1^+/+^, TGIF1^+/-^ and TGIF1^−/−^ animals. Left panel magnification x40 and right panel magnification x200 as indicated. (B) Number of surviving crypts measured in small intestinal sections 3 days after sham or 19 Gy localized small intestinal irradiation in wild type, TGIF1^+/+^, TGIF1^+/−^ and TGIF1^−/−^ animals. (C) Villus height measured in small intestinal sections 3 days after sham or 19 Gy localized small intestinal irradiation in wild type, TGIF1^+/+^, TGIF1^+/−^ and TGIF1^−/−^ animals. The hatched bars represent the wild type control values. *p<0.001 compared to sham animals; ^#^p<0.005 and ^##^p<0.001 between irradiated groups. 3<n<6 per group.
(TIF)

Table S1 **List of Pre-developped TaqMan gene expression assay used to quantify transcripts levels.**
(TIF)
