# Supplementary figures and images for: The TGF-β/Smad Repressor TG-Interacting Factor 1 (TGIF1) Plays a Role in Radiation-Induced Intestinal Injury Independently of a Smad Signaling Pathway
Source: PLoS One. 2012 May 2;7(5):e35672. doi: 10.1371/journal.pone.0035672 (PMC3342305; doi:10.1371/journal.pone.0035672)

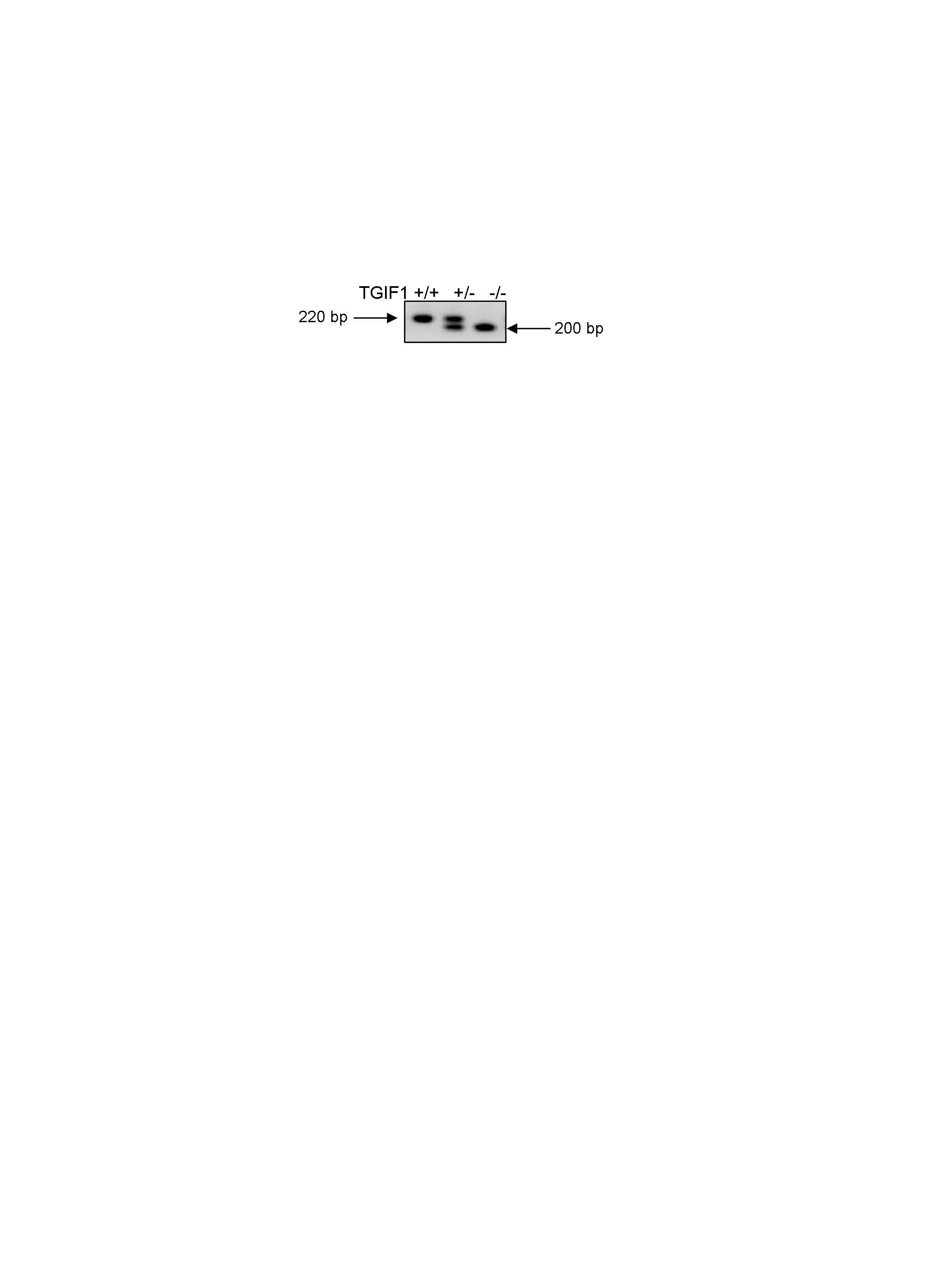

Supplement: Figure S1 — Example of PCR screening for TGIF1 genotype. (TIF) [file pone.0035672.s001.tif]

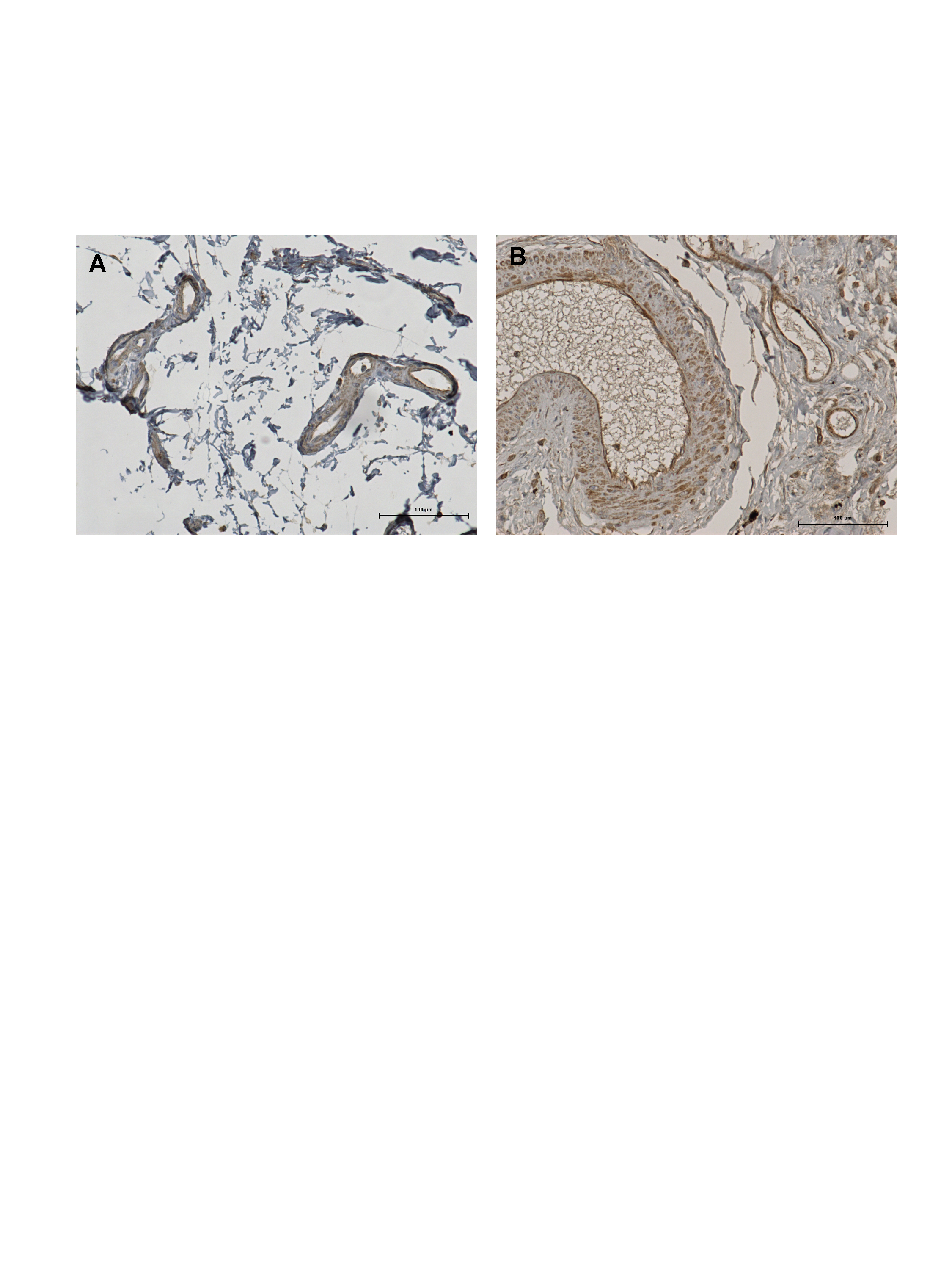

Supplement: Figure S2 — Representative microscopic images from immuno-labelling of TGIF1 on human rectal tissue submucosal vessels. (A) unirradiated tissue; (B) dystrophic vessel in the irradiated area from the same patient. Magnification x 200. (TIF) [file pone.0035672.s002.tif]

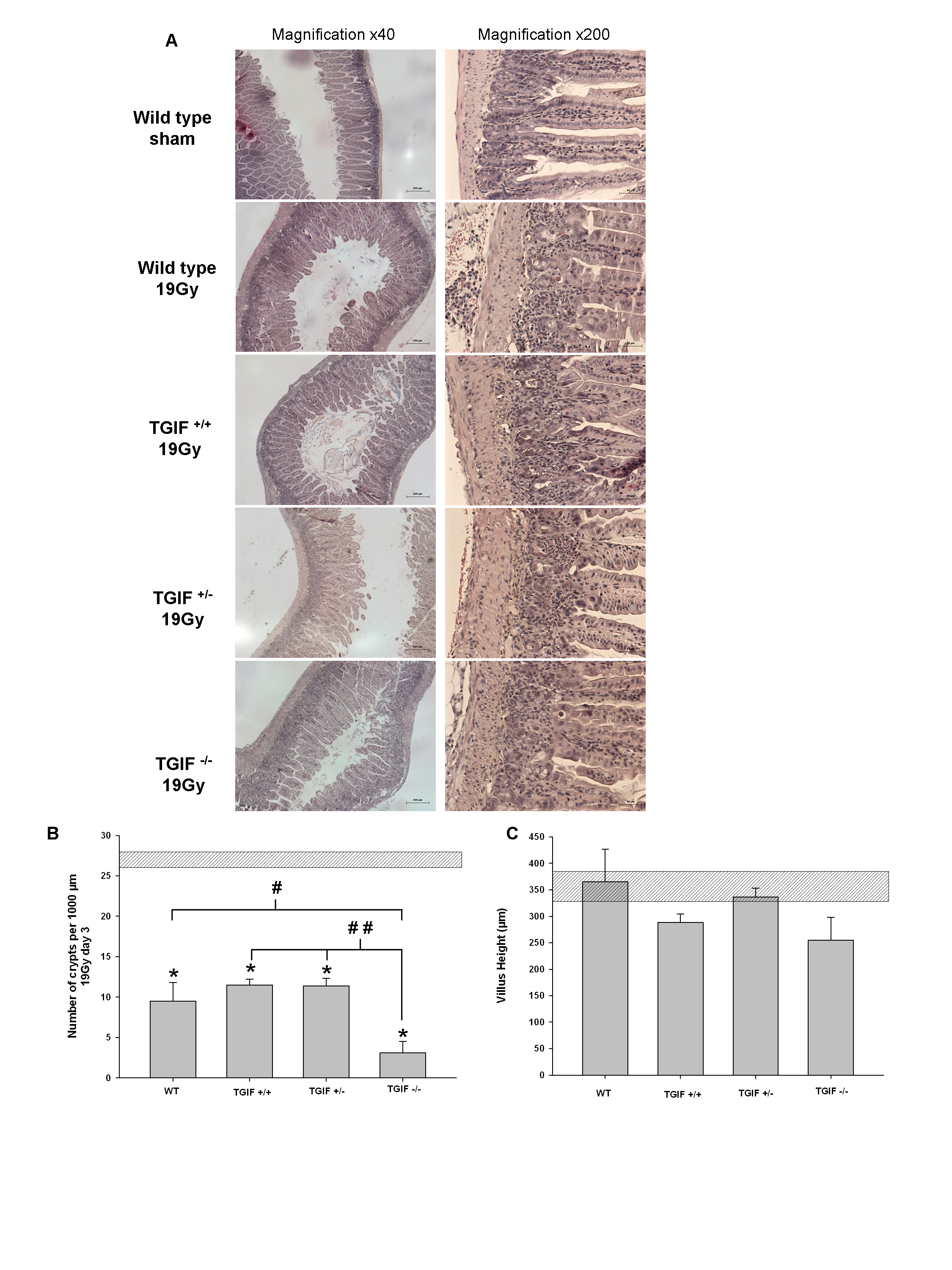

Supplement: Figure S3 — Radiation-induced small intestinal damage. (A) Images of small intestinal tissues obtained 3 days after sham or 19 Gy localized small intestinal irradiation in wild type, TGIF1+/+, TGIF1+/- and TGIF1−/− animals. Left panel magnification x40 and right panel magnification x200 as indicated. (B) Number of surviving crypts measured in small intestinal sections 3 days after sham or 19 Gy localized small intestinal irradiation in wild type, TGIF1+/+, TGIF1+/− and TGIF1−/− animals. (C) Villus height measured in small intestinal sections 3 days after sham or 19 Gy localized small intestinal irradiation in wild type, TGIF1+/+, TGIF1+/− and TGIF1−/− animals. The hatched bars represent the wild type control values. *p<0.001 compared to sham animals; #p<0.005 and ##p<0.001 between irradiated groups. 3<n<6 per group. (TIF) [file pone.0035672.s003.tif]

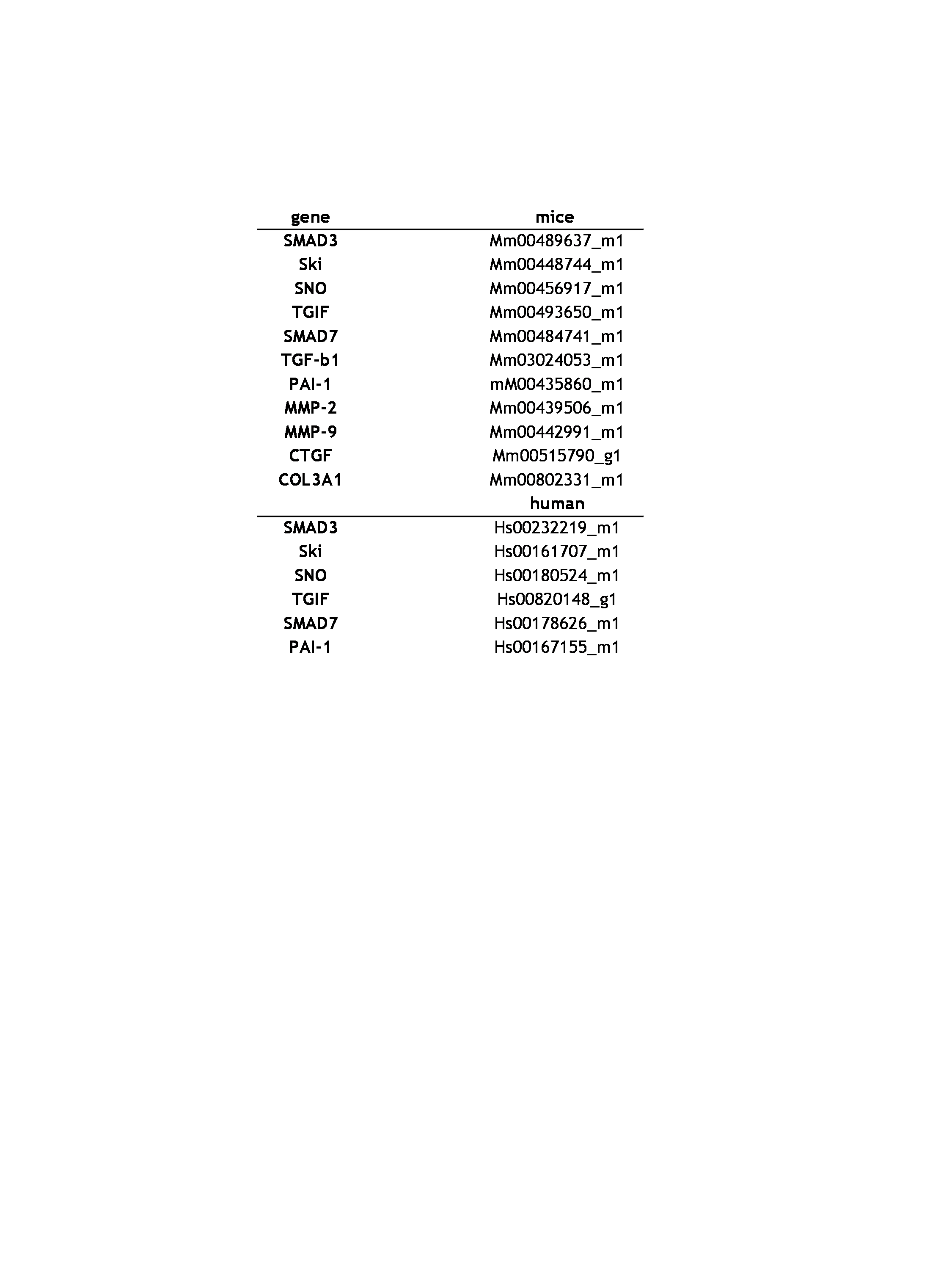

Supplement: Table S1 — List of Pre-developped TaqMan gene expression assay used to quantify transcripts levels. (TIF) [file pone.0035672.s004.tif]
